# Cardiac Magnetic Resonance Imaging in Diagnostics and Cardiovascular Risk Assessment

**DOI:** 10.3390/diagnostics15020178

**Published:** 2025-01-14

**Authors:** Patrycja S. Matusik, Katarzyna Mikrut, Amira Bryll, Tadeusz J. Popiela, Paweł T. Matusik

**Affiliations:** 1 Department of Radiology, Faculty of Medicine, Jagiellonian University Medical College, 31-008 Kraków, Poland; patrycja.s.matusik@gmail.com (P.S.M.); bryllamira@gmail.com (A.B.); msjpopie@cyf-kr.edu.pl (T.J.P.); 2 Department of Diagnostic Imaging, University Hospital, 30-688 Kraków, Poland; 3 Department of Cardiology, Advocate Lutheran General Hospital, Park Ridge, IL 60068, USA; katie.mikrut@gmail.com; 4 Department of Electrocardiology, Institute of Cardiology, Faculty of Medicine, Jagiellonian University Medical College, 31-008 Kraków, Poland; 5 Department of Electrocardiology, St. John Paul II Hospital, 31-202 Kraków, Poland

**Keywords:** cardiac magnetic resonance, CMR, cardiovascular risk stratification, LVM indexing, LGE, novel techniques, personalized medicine

## Abstract

Cardiac magnetic resonance (CMR) allows for analysis of cardiac function and myocardial tissue characterization. Increased left ventricular mass (LVM) is an independent predictor of cardiovascular events; however, the diagnosis of left ventricular hypertrophy and its prognostic value strongly depend on the LVM indexation method. Evaluation of the quantity and distribution of late gadolinium enhancement assists in clinical decisions on diagnosis, cardiovascular assessment, and interventions, including the placement of cardiac implantable electronic devices and the choice of an optimal procedural approach. Novel CMR techniques, such as T1 and T2 mapping, may be used for the longitudinal follow-up of myocardial fibrosis and myocardial edema or inflammation in different groups of patients, including patients with systemic sclerosis, myocarditis, cardiac sarcoidosis, amyloidosis, and both ischemic and non-ischemic cardiomyopathy, among others. Moreover, CMR tagging and feature tracking techniques might improve cardiovascular risk stratification in patients with different etiologies of left ventricular dysfunction. This review summarizes the knowledge about the current role of CMR in diagnostics and cardiovascular risk assessment to enable more personalized approach in clinical decision making.

## 1. Introduction

Cardiac magnetic resonance (CMR) is the gold standard method for assessment of left ventricular mass (LVM) and volumes and enables analysis of cardiac function and myocardial tissue composition [1,2]. It provides important markers for early diagnosis of cardiovascular disorders and may be combined with other clinical data to improve risk stratification [1,3]. Among other things, CMR permits detection of potential substrates for life-threatening arrhythmias, enables assessment of microvascular damage, and may exclude significant myocardial abnormalities, providing valuable clinical information [4,5,6]. The prognostic importance of this modality has been demonstrated in a wide range of patients, including those with coronary artery disease (CAD), cardiomyopathies, and heart failure (HF) [4,5,7,8,9,10,11,12,13,14].

In this review, we summarize current knowledge about the role of different CMR parameters in diagnostics and prediction of cardiovascular events, as well as their impact on a more personalized approach to clinical decision making.

## 2. Prognostic Value of Left Ventricular Hypertrophy Type

**Figure 1:**
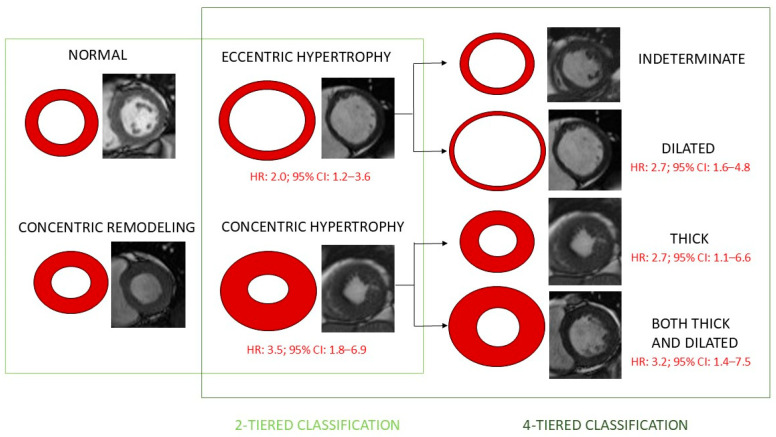
Left ventricular remodeling and comparison of two-tiered and four-tiered classifications of left ventricular hypertrophy regarding all-cause mortality. Based on refs. [19,20,21]. Legend: When using the conventional two-group classification system, both eccentric and concentric left ventricular hypertrophy predicted all-cause mortality in a multivariable Cox regression analysis. When using the four-group classification system, eccentric dilated left ventricular hypertrophy and both concentric nondilated and dilated LVH were associated with increased risk for all-cause mortality, but indeterminate LVH was not associated with this outcome. Indeterminate left ventricular hypertrophy is related to not meeting criteria for increased concentricity^0.67^ and increased left ventricular end-diastolic volume/body surface area.

Increased LVM and left ventricular hypertrophy (LVH) are independent predictors of cardiovascular events [15,16,17,18]. Three kinds of abnormal left ventricular (LV) remodeling, namely, concentric remodeling, concentric hypertrophy, and eccentric hypertrophy were distinguished among patients with hypertension [19]. This categorization was based on LVM and the ratio of LV wall thickness to LV chamber size and is related to the risk of cardiovascular events [17]. However, some studies suggest that the LV remodeling results from independent changes in LV wall thickness and LV diameter and propose four-tiered categorization [19,20,21]. This classification of LVH is based on whether LV wall thickness and LV diameter are increased. The comparison of these two classifications and their associations with all-cause mortality are depicted in Figure 1.

Concentric remodeling is the most frequent LV geometric abnormality and may affect about 9.4% of the general population investigated by echocardiography [22]. This is followed by eccentric non-dilated LVH at 6.3%, concentric LVH at 4.6%, and eccentric dilated LVH at 3.5% [22]. Concentric remodeling (normal LVM with an increased thickness/diameter ratio) is related to a higher risk for the above adverse outcomes than normal LVM with a normal wall thickness to diameter ratio [17]. Concentric LVH (increased LVM and LV wall thickness/diameter ratio) is linked with higher risk for all-cause mortality, cardiovascular death, and death or heart failure hospitalization when compared to eccentric hypertrophy (increased LVM and low LV wall thickness/diameter ratio). Importantly, both the type and extent of LVH as well as local functional abnormalities have prognostic value [17,23]. One observational multicenter study showed that patients who died during the median follow-up time (2.4 years; interquartile range: 1.2–2.9 years) had, among other things, lower left ventricular ejection fraction (LVEFs) and higher wall motion score [23].

The potential prognostic significance of changes in LV geometry or the LVM index may be valuable for clinical practice, including assessment of reverse cardiac remodeling in hypertensive patients undergoing treatment [24]. LVH indicates structural and functional myocardial alteration and is linked to a worse prognosis. Therefore, echocardiography is recommended in patients with ECG abnormalities (ECG should be analyzed for LVH) or when there are signs/symptoms of cardiac disease in hypertensive patients [25]. Monitoring changes in LV geometry and the LVM index during treatment can indicate treatment effectiveness and the risk associated with potentially irreversible myocardial damage. Repeated assessment may help identify patients who do not respond well to treatment (those with persistent LVH), allowing for more intensive management to mitigate cardiovascular risk. Importantly, LVH can require different treatment strategies based on its type and etiology [26]. Concentric LVH results from chronic pressure overload (e.g., hypertension, aortic stenosis), leading to uniform myocardial thickening and reduced cavity size. Treatment focuses on tight blood pressure control and addressing diastolic dysfunction to prevent heart failure or potential aortic valve replacement in the case of severe aortic valve stenosis [27]. It should be mentioned that hypertension may be associated with either concentric or eccentric LVH [28]. However, eccentric LVH is frequently associated with volume overload (e.g., mitral regurgitation, dilated cardiomyopathy (DCM)), causing ventricular dilatation. Management includes careful diuretic use to control symptoms, valve repair/replacement as a causal treatment in patients with significant valve disease, and disease-modifying agents to reduce remodeling or improve prognosis. Anticoagulation and antiarrhythmic strategies are often necessary in patients with concomitant atrial fibrillation [29]. Systemic conditions like amyloidosis or Fabry disease may also cause LVH and require disease-specific treatments. Therefore, understanding the LVH type and etiology may be valuable for tailoring treatment, optimizing outcomes, and mitigating associated risks. Table 1 shows selected clinical conditions in which LVH is valuable in diagnostics and risk stratification [24,25,26,27,30,31,32,33].

## 3. The Indexing Process of LVM in Cardiovascular Risk Stratification

Despite the important prognostic significance of LVM, there are still controversies surrounding its measurement and indexing. The confounding effects of body size on LVM calculation and the need to index LVM have been emphasized [34]. Different methods of normalizing LVM have been investigated. However, the best method of LVM indexation has not been clearly established [35,36,37,38,39]. A comparison of scaling methods used for indexing of LVM is summarized and depicted in Table 2.

Depending on the algorithm used for LVM measurement and indexation, there are various cut-off values for LVH diagnosis with different levels of accuracy [37,38,43]. Studies that have been performed so far highlight that depending on the method of LVM indexing, the prevalence of hypertrophy is different [44,45]. Table 3 demonstrates a selection of the main studies investigating an indexed LVM during CMR imaging in a healthy population [16,34,38,41,42,46,47,48]. Examples of longitudinal studies using CMR to estimate LVM as predictor of clinical outcomes have also demonstrated that the method of indexing LVM may influence the ability to predict future cardiovascular events [34]. LVH defined by LVM/height^1.7^ had the strongest association with cardiovascular events and all-cause mortality in adults free of overt cardiovascular disease [34]. These data suggest that the choice of method for indexing LVM may have important influence on LVH diagnosis and emphasize the role the LVM indexing method plays in LVH diagnosis, cardiovascular risk assessment, and clinical management.

Currently, normalization of LVM for body surface area (BSA) is the recommended standard approach in clinical evaluation with use of both echocardiography and CMR [34,37,39,41]. Recently, Luu et al. derived sex- and age-specific normal reference values for LVM indexed to BSA based on the largest multi-ethnic population to date, which consisted of 3206 participants free of cardiovascular disease [47]. Future studies investigating the associations of LVH diagnosis with these novel reference values are encouraged.

## 4. Importance of Standard CMR Parameters in Cardiovascular Risk Assessment

### 4.1. Cardiac Function Parameters Assessed in CMR and Risk Prediction

**Figure 2:**
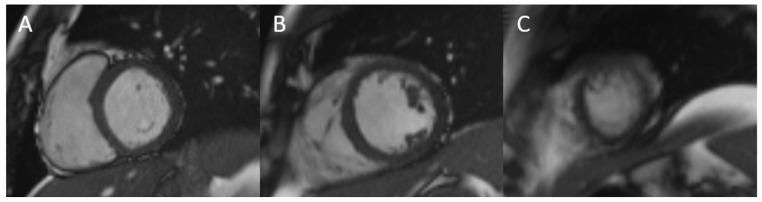
Basal (**A**), mid (**B**), and apical (**C**) short-axis cine images used for the evaluation of cardiac function and structure.

LVEF is one of the most commonly used cardiac imaging parameters in clinical practice and should be reported especially in the context of diagnostics of HF. Currently, it is also the main factor used for clinical sudden cardiac death (SCD) risk stratification. Measurement of LVEF (Figure 2), despite limitations associated with load dependency and reproducibility, has been shown to relate to cardiovascular risk in different groups of patients [49,50]. It was demonstrated that in patients with stable CAD, LVEF thresholds of ≤50 and ≤35%, with simultaneous evaluation of the presence of late gadolinium enhancement (LGE), permit for assessment of risk of cardiac death and non-fatal myocardial infarction [49]. What is more, in patients with LV noncompaction, LVEF was the variable most strongly associated with major adverse cardiovascular events (MACE), defined as HF hospitalization, ventricular arrhythmias, systemic embolisms, or all-cause mortality [50]. However, it should be emphasized that addition of other CMR findings, such as LGE, to the risk assessment provides more comprehensive evaluation [4].

LV end-diastolic volume (LVEDV), LV end-systolic volume (LVESV), and LV stroke volume (LVSV) are also useful in cardiac risk assessment across various patient populations [51]. LVEDV is an important indicator of ventricular dilatation, often seen in HF patients. Increased LVEDV in HF patients suggests adverse remodeling and is associated with worse clinical outcomes [52]. After acute myocardial infarction (AMI), high LVEDV can signal poor prognosis and a higher risk of future HF or recurrent events [51]. In hypertensive and CAD patients, increased LVEDV may indicate early signs of ventricular remodeling, necessitating closer monitoring and risk management. LVESV is a strong prognostic marker in HF, reflecting impaired systolic function. Reductions in LVESV with treatment are linked to improved outcomes, while persistently high LVESV is associated with increased mortality and arrhythmia risk in DCM [53]. LVSV, the difference between LVEDV and LVESV, represents the amount of blood ejected during each heartbeat. It is a key determinant of cardiac output, with reduced LVSV indicating compromised cardiac function, frequently observed in HF and cardiomyopathies. Low LVSV correlates with reduced exercise tolerance and poor functional status, among other things, in patients with valvular disease or CAD, and serves as a predictor of adverse events [54]. Monitoring changes in these parameters helps to guide therapeutic decisions, assess treatment responses, or evaluate disease progression, making them helpful in the management of various cardiovascular conditions.

### 4.2. Parameters Allowing for Tissue Characterization in CMR and Risk Prediction

There are different methods of LGE assessment—two-standard deviation (SD), three-SD, and full-width half maximum (FWHM)—each uniquely impacts cardiovascular risk stratification by quantifying myocardial scarring and fibrosis [55]. The 2-SD method is sensitive, detecting subtle, low-density fibrosis, useful for early disease detection, such as in hypertrophic cardiomyopathy (HCM) or DCM, where even small fibrotic changes indicate disease progression [56]. However, it may overestimate fibrosis by capturing noise or minor artifacts. The 3-SD method is more specific, identifying pronounced scarring, which is closely linked to arrhythmic risk and SCD. It is effective in patients with larger scars, like those post-MI, but may miss diffuse fibrosis. FWHM sets a threshold at 50% of peak signal intensity, providing balanced sensitivity and specificity, especially in detecting transmural infarcts that predict arrhythmias and poor outcomes [57]. However, FWHM may be less effective for diffuse fibrosis, such as in myocarditis. The choice of method affects risk assessment; however, generally larger scar burdens are consistently linked to higher risks of arrhythmias, HF, and increased mortality.

**Figure 3:**
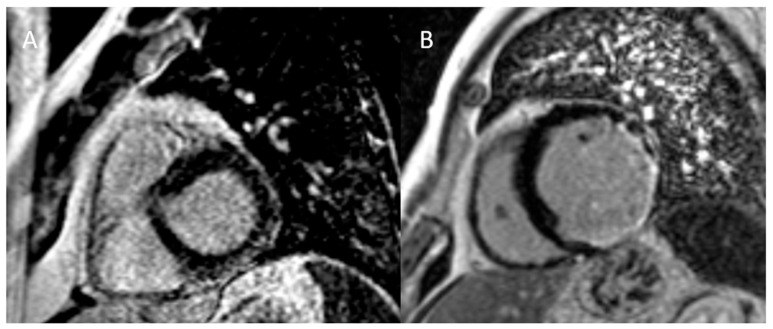
Subepicardial late gadolinium pattern in a patient with myocarditis (**A**) and subendocardial/transmural late gadolinium enhancement in a patient with myocardial infarction (**B**).

LGE patterns provide valuable insights into myocardial pathology (Figure 3) [58]. A subendocardial or transmural LGE pattern suggests ischemic damage, commonly due to myocardial infarction, while mid-wall LGE in the interventricular septum is often seen in nonischemic cardiomyopathies like DCM or myocarditis. Epicardial LGE may be associated with inflammatory conditions such as myocarditis or sarcoidosis. Patchy or diffuse LGE may indicate infiltrative diseases like amyloidosis or sarcoidosis, while LGE at the right ventricular insertion points is typically found in HCM or pulmonary hypertension [58].

CMR allows for detection of potential substrates for life-threatening arrhythmias [4]. It was demonstrated that presence of LGE is related to ventricular arrhythmias both in patients with LVEF ≤ 35% and in patients with LVEF > 35% [4]. CMR may also play a role in cardiovascular risk stratification in oncological patients [59]. Assessment of LGE during CMR could improve risk assessment in various clinical scenarios [58,60]. It was demonstrated that both the presence and the extent of LGE have diagnostic and prognostic value in patients with cardiomyopathies of both ischemic and non-ischemic etiology [61,62,63,64]. Patients with multiple areas of LGE have a higher risk of cardiac events (heart failure death or hospitalization for worsening heart failure, SCD, and aborted SCD defined as non-fatal ventricular fibrillation, sustained ventricular tachycardia, or adequate implantable cardioverter–defibrillator therapies) and worse reverse LV remodeling [65]. Currently, evaluation of LGE is part of the SCD risk assessment in the 2020 American College of Cardiology (ACC)/American Heart Association (AHA) HCM guidelines [66].

Quantitative analysis of LGE by CMR has prognostic value in SCD prediction, independent of baseline characteristics in patients with HCM [67]. In this group of patients, extensive areas of LGE, especially those representing ≥15% of LVM, have been shown to be associated with an increased risk of SCD [14]. Moreover, it was found that the amount of LGE might outperform the European HCM Risk-SCD score and the American College of Cardiology Foundation (ACCF)/AHA algorithm in SCD risk assessment in a subgroup of patients with HCM [60].

In patients with DCM, there was a trend towards an increased risk of adverse clinical events already in LGE areas affecting >4.8% of LVM [5]. In addition, it has been observed that in about 35% of DCM patients myocardial fibrosis presence (most often in the basal anterolateral segment) is a predictor of worse outcomes like overall mortality or hospitalization due to cardiovascular events [7]. Therefore, the addition of LGE assessment to imaging protocols in at least subgroups of patients may significantly improve risk stratification.

Among patients with CAD, which is frequent in patients with left ventricular dysfunction and HF [68], CMR may be useful for evaluation of myocardial viability and guiding revascularization [69]. LGE may help to distinguish viable from non-viable myocardium by indicating scar tissue, while cine imaging and stress CMR may assist in assessment of wall motion and contractile reserve, indicating potential myocardial recovery. T1 mapping and extracellular volume (ECV) quantify diffuse fibrosis, and T2 mapping detects myocardial edema, revealing areas of potentially salvageable tissue. Microvascular obstruction (MVO), often visible in post-MI patients, suggests severe myocardial damage and poorer myocardial recovery [70]. Importantly, infarct size and the presence of MVO strongly predict cardiovascular events and mortality [63]. It has also been shown that in patients with a history of MI, the size of the peri-infarct zone measured with CMR may predict SCD [64]. Additionally, the presence of LGE in patients with clinical suspicion of CAD and previously undiagnosed MI is a predictor of cardiovascular events, including SCD [13]. However, it should be noted that potential concerns regarding gadolinium-based contrast administration in patients with severe renal dysfunction should be balanced by the increased risk observed in these patients at the same time [29,71].

It is important to recognize that LGE can highlight not only myocardial but also pericardial involvement, which carries clinical implications [72]. LGE in pericardium, often associated with conditions like chronic pericarditis or post-surgical inflammation, may indicate ongoing inflammation or fibrosis, influencing patient management and long-term outcomes. LGE in pericardium is frequently found in patients years after cardiac surgery, with up to 44% of them showing this feature. Although no patients displayed symptoms or required pericarditis treatment, LGE was more prevalent with multiple surgeries. Among those who underwent biopsies, all showed pericardial fibrosis, and nearly half with significant LGE had low-grade inflammation. These findings suggest that pericardial LGE may reflect chronic, subclinical inflammation post-surgery rather than active pericarditis [72].

**Figure 4:**
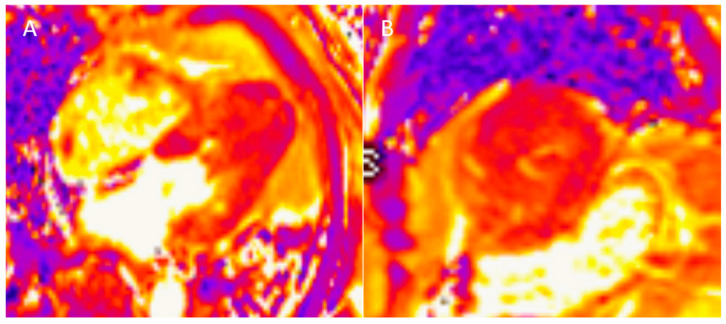
Abnormal T2 mapping in a patient with hypertrophic cardiomyopathy (**A**) long-axis, (**B**) short-axis.

**Figure 5:**
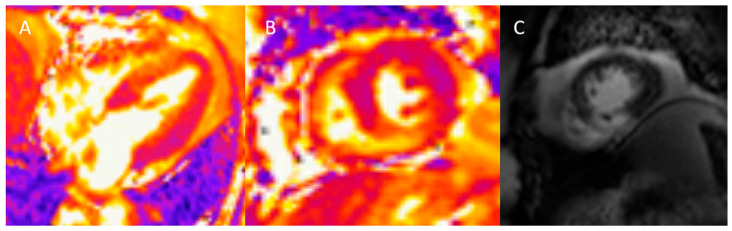
Abnormal T2 mapping: (**A**) long-axis, (**B**) short-axis, and (**C**) microvascular obstruction (short-axis) in a patient with myocardial infarction.

Recently, several novel techniques used in CMR have been described. The addition of T1 or T2 preparation pulses (“mapping”) provides additional tissue characterization (Figure 4 and Figure 5) [73]. While LGE is limited in the detection of diffuse fibrosis, the mapping techniques make that assessment possible [65]. Moreover, T1 mapping and ECV calculation may also depict microscopic fibrosis in the absence of LGE [74]. Importantly, native T1 mapping and ECV may be used for the longitudinal follow-up of increasing myocardial fibrosis in different groups of patients, including patients with systemic sclerosis and amyloidosis, as well as both ischemic and non-ischemic cardiomyopathy [75,76,77,78]. It was demonstrated that higher native T1 values are associated with arrhythmic endpoints in patients with systemic sclerosis [75]. It was also shown that measurement of native T1 and ECV may be useful for the risk stratification of patients with cardiac amyloidosis [76]. In a recent study concerning this group of patients, it was demonstrated that native T1 values > 1044 ms and ECV values > 45% were associated with higher probability of death [68]. Moreover, in a study performed on patients with non-ischemic cardiomyopathy, ECV provided incremental prognostic association with heart failure outcomes over native T1 mapping or LGE, assessed individually [77]. Similarly, a correlation between ECV and cardiac events in a general patient cohort has been emphasized [78]. Native T1 mapping can be derived without the need for gadolinium-based contrast agent administration and may be useful for patients in whom the administration of contrast agents is contraindicated [73]. T2-weighted imaging can be helpful to visualize myocardial edema, which in patients with ST-elevation myocardial infarction (STEMI) represents the extent of the myocardium or area at risk [79]. However, the current limitation of this method is the lack of standardization of mapping and post-processing techniques.

In cardiomyopathies, CMR enables detailed assessment of myocardial structure and function. Cine imaging allows for measurement of volumes and LVEF, while LGE identifies fibrosis, which is crucial for risk stratification in HCM and DCM [80,81]. T2 mapping detects edema in myocarditis, while T1 mapping with ECV quantifies infiltration in conditions like amyloidosis [82]. T2* mapping suggests hemorrhage and MVO, particularly in patients with ischemia, to predict outcomes. Collectively, these parameters make CMR a non-invasive, multi-faceted tool for accurate diagnosis, risk evaluation, and personalized management across a spectrum of cardiac conditions and help to exclude cardiac structural abnormalities when a clinical picture suggests primary electrical disease [6].

In a study of 405 patients undergoing CMR for suspected myocarditis, those with a normal CMR (defined by normal LV volumes, normal LVEF, and absence of LGE) demonstrated a favorable long-term prognosis, regardless of clinical symptoms [82]. Over a median follow-up of approximately 4.4 years, the overall mortality was 3.2%, with all cardiac deaths, SCD, and ICD shocks occurring in patients with abnormal CMR findings. A significantly lower risk of MACE in patients with normal CMR highlights the prognostic value of CMR in risk stratification.

CMR may be used for assessment of patients with AMI, particularly in cases of myocardial infarction with non-obstructive coronary arteries (MINOCA) [83]. CMR provides detailed imaging of myocardial structure and tissue characteristics, indicating potential causes like myocardial infarction, myocarditis, or takotsubo cardiomyopathy [84]. Techniques such as LGE and T1/T2 mapping help differentiate infarct patterns from non-ischemic injuries, aiding in diagnosis, risk stratification, and management, ultimately improving outcomes in MINOCA.

Bergamaschi et al. in their study highlight the prognostic significance of T2 mapping in evaluating myocardial edema in patients with STEMI who underwent primary percutaneous coronary intervention [85]. Higher T2 values in the non-infarcted myocardium (NIM) (above 45 ms) were associated with larger infarct size, the presence of microvascular obstruction, and left ventricular dysfunction. Notably, these elevated NIM T2 values did not correlate with systemic inflammatory markers such as C-reactive protein or white blood cell count, nor with T2 values in non-cardiac tissues (pectoralis muscle, liver, or spleen). This specificity to the myocardium suggests that T2 mapping could serve as a targeted biomarker for post-MI myocardial injury. At a 17-month follow-up, higher T2 values in NIM (>45 ms) were associated with a significantly increased risk of MACE, particularly a higher rate of myocardial reinfarction (26.3% vs. 1.4% in the remainder patients). In multivariable analysis, higher NIM T2 values, independently of other risk factors, predicted MACE with a hazard ratio of 2.8, indicating that increased myocardial edema post-STEMI strongly correlates with poorer outcomes. These may identify patients who may be prone to reinfarction and other adverse events, allowing improved risk stratification of patients and potentially adjustment of therapeutic approaches.

Carrick et al. showed that early post-MI native T1 values in the infarct core were significantly associated with N-terminal pro-B-type natriuretic peptide (NT-proBNP) levels at 6 months, indicating LV remodeling, independent of baseline LV volume [86]. Elevated infarct core T1 was linked with adverse remodeling, increased NT-proBNP, and higher risks of all-cause mortality or HF hospitalization during follow-up. Compared to T2 or T2* mapping for hemorrhage and MVO, infarct core T1 was more consistently correlated with LV outcomes and cardiac events. This study supports native T1 mapping as a valuable prognostic tool post-STEMI and highlights MVO’s continued importance in predicting clinical outcomes.

Mapping techniques might also be used in other organs. Interestingly, hepatic T1 mapping may play a role as a novel cardio-hepatic axis imaging biomarker early after STEMI. The hepatic response after STEMI may be associated with increased mortality and morbidity. Bergamaschi et al. [87] evaluated hepatic native T1 as an indicator of right ventricular (RV) involvement after STEMI. In 133 STEMI patients post-primary angioplasty, higher hepatic T1 values (>605 ms) were associated with RV dysfunction, including a lower RV ejection fraction (RVEF), increased RV end-diastolic volume index (RVEDVi), and the presence of LGE in RV. Patients with elevated hepatic T1 also had significantly higher NT-proBNP levels, suggesting RV strain. RVEDVi and RVEF were independent predictors of increased hepatic T1. These findings suggest that RV involvement post-STEMI leads to hepatic congestion, reflected by elevated hepatic T1 values. 

Selected studies describing associations between CMR parameters and cardiovascular events are depicted in Table 4.

**Figure 6:**
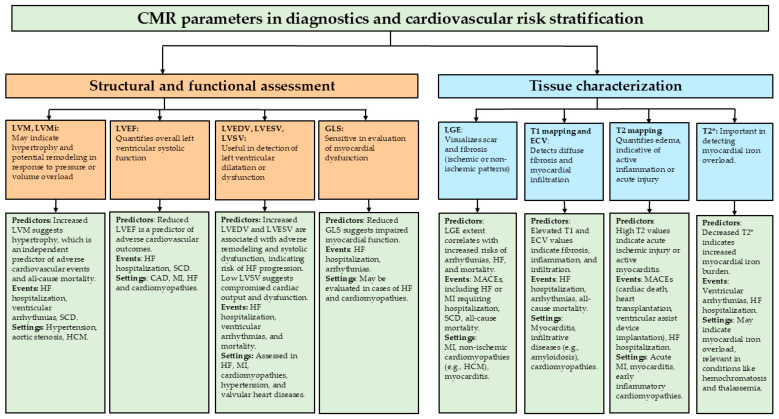
Characteristics of myocardial structure and function along with tissue characterization in cardiac magnetic resonance combined with their relevance to potential predictors, possible clinical events, and settings. Abbreviations: CAD—coronary artery disease; CMR—cardiac magnetic resonance; ECV—extracellular volume; GLS—global longitudinal strain; HCM—hypertrophic cardiomyopathy; HF—heart failure; LGE—late gadolinium enhancement; LVEF—left ventricular ejection fraction; LVEDV—left ventricular end-diastolic diameter; LVESV—left ventricular end-systolic diameter; LVM—left ventricular mass; LVMI—left ventricular mass indexing; LVSV—left ventricular stroke volume; MACE—major adverse cardiovascular events; MI—myocardial infarction; SCD—sudden cardiac death.

A summary of the assessment of CMR parameters in diagnostics and cardiovascular risk stratification is depicted in Figure 6.

### 4.3. Role of CMR in Cardiovascular Procedures

Assessment of myocardial scar presence and localization may be valuable before cardiovascular procedures. Different parameters may be evaluated, such as the LGE, LGE mass, percentage of LGE, infarct transmurality, and peri-infarct zone [73]. All of those parameters were reported to be associated with the occurrence of ventricular arrhythmias or SCD [13,90,91,92,93].

It has been shown that, in patients with LVEF > 30%, significant scarring (>5% of LV) is related to a high risk of death or discharge of an implantable cardioverter–defibrillator (ICD) due to ventricular tachycardia or ventricular fibrillation, comparable to patients with LVEF ≤ 30% [94]. In contrast, in patients with LVEF ≤ 30%, the absence or presence of only minimal scars identified a cohort at low risk of death or ICD discharge. It was also shown that among patients with DCM and LVEF ≤ 35%, implantation of an ICD was associated with a reduction in mortality only among those with the presence of LGE in the CMR study [95]. It has been proven that the presence of a transmural LGE in the posterolateral LV wall is related to reduced response to conventional cardiac resynchronization therapy (CRT) [96]. Therefore, assessment of LGE before the procedure may be useful and may in some cases indicate patients for whom physiologic pacing could be a better alternative.

Interestingly, combined myocardium tissue heterogeneity and interstitial fibrosis assessed by native T1 mapping is a predictor of ventricular tachycardia and ventricular fibrillation both in ischemic and non-ischemic cardiomyopathies [97,98]. This method might be used to improve risk stratification and to schedule patients for ICD placement in primary prevention in nonischemic HF patients in whom gadolinium contrast is contraindicated.

## 5. Stress CMR and Cardiovascular Risk Prediction

Several large studies have shown the prognostic value of stress CMR [89,99,100,101]. This method adds long-term prognostic value to mortality prediction when compared to conventional risk factors [89]. In patients with a first inconclusive test in diagnostics of CAD (exercise electrocardiogram, stress echocardiography or single photon emission computed tomography), stress CMR may have additional prognostic value to predict MACE when compared to other cardiovascular risk factors [101]. In a multicenter cohort of patients with stable chest pain syndromes, stress CMR was performed and demonstrated that patients without CMR ischemia or LGE had a low incidence of cardiac events and decreased need for coronary revascularization [100]. On the other hand, it was shown that coronary revascularization performed within 90 days after CMR (CMR-related revascularization) was related to a reduced mortality in patients with severe ischemia [89]. Moreover, another study performed in a heterogenous population of patients with known or suspected CAD also showed that stress CMR has prognostic value in the prediction of patient’s mortality [99].

## 6. Novel Techniques in CMR in Cardiovascular Risk Stratification

### 6.1. Diffusion Tensor Imaging

Diffusion tensor imaging (DTI) is a novel technique that provides insight into the myocardial fiber architecture and has shown promise in detecting early microstructural changes linked to adverse outcomes [102]. DTI has clinical and research applications, particularly in understanding myocardial mechanics and detecting microstructural changes in diseases such as HCM or DCM [103]. It may also help identify subjects at risk of developing HF, not only in the context of cardiomyopathy, but also in patients with congenital and ischemic heart disease. It can identify disruptions in fiber alignment post-myocardial infarction, aiding in the assessment of remodeling and predicting outcomes [102,103]. Abnormal myofiber orientation detected by DTI has been associated with an elevated risk for cardiovascular events, including arrhythmias and HF progression. However, challenges such as long acquisition times, susceptibility to motion artifacts, and the need for specialized analysis remain [103]. Despite these hurdles, DTI has the potential to aid clinical diagnosis and risk assessment through microscopic phenotyping.

### 6.2. Strain Imaging

It has been demonstrated that both focal and diffuse fibrosis cause myocardial stiffness, which can be assessed using strain imaging [74]. Not all abnormal strain is caused by fibrosis; therefore, we should not consider abnormal strain as a specific marker of fibrosis. Strain imaging evaluates myocardial deformation during contraction and relaxation. It has been demonstrated that impaired strain is associated with adverse outcomes [74]. Myocardial strain measures how the heart muscle deforms and is considered a more sensitive indicator of heart disease than traditional ejection fraction metrics, especially at early stages of myocardial dysfunction development [104]. Myocardial strain can be assessed by different methods during a CMR scan. Myocardial tagging is directly measured by myocardial tissue properties but requires extra acquisition time and time-consuming post-processing [105]. Feature tracking is a post-processing technique in which additional acquisition time is not required, and it is easier to perform. Both myocardial tagging and feature tracking techniques may be helpful in the assessment of cardiovascular risk [88,106,107,108]. It has been demonstrated that abnormalities in circumferential strain, assessed with the tagging method, are associated with inducible ventricular arrhythmias and identification of the grey zone [106,107]. Moreover, in a large population, global circumferential strain, in addition to clinical variables like LVEF and LGE, had independent prognostic value for MACE defined as all-cause mortality, hospitalization due to HF exacerbation, and SCD [88]. Similarly, global longitudinal strain measurement assessed by the feature tracking technique is a risk marker for ventricular arrhythmias and SCD [108]. However, larger studies are needed to confirm those findings. There is lack of standardization for some strain assessment methods, which makes it challenging to implement them in clinical practice [74].

## 7. Limitations

CMR is valuable for diagnosing diseases of the heart but has limitations [109]. General limitations include contraindications in patients with severe renal disease due to the risk of nephrogenic systemic fibrosis from gadolinium-based contrast agents; however, it should be noted that the risk is very low with newer agents. Claustrophobia and anxiety are other challenges which may be encountered, especially during long scans, sometimes necessitating sedation. Patients with non-MRI-compatible implants may also limit clinical use of CMR [109]. Importantly, implanted devices may also cause artifacts which can decrease image quality and limit assessment or require specific CMR protocols. Additionally, the duration of CMR exams, which can last 30–90 min, poses challenges, especially in acute settings. Therefore, development and application of time-effective CMR scanning protocols is desirable. Specific technique limitations include the potential inability of LGE to detect diffuse fibrosis and its dependency on gadolinium, limiting its use in cases of renal insufficiency. LGE assessment is also prone to image artifacts, particularly in thin-walled structures and patients with arrhythmias. T1 and T2 mapping are prone to variability in heart rates and scanner types, as well as protocol differences, with limited data on normal reference ranges and a risk of misinterpreting high native T1 values [110]. T2 imaging is sensitive to motion artifacts and may yield false positives, lacking specificity for differentiating edema causes. General image quality can be affected by artifacts resulting from arrhythmias, respiratory motion, or a high body mass index, impacting the reliability of functional measurements. Moreover, optimal contrast timing for LGE may be difficult in routine practice. Finally, cost and accessibility are other barriers, as CMR is more expensive and less available than other imaging modalities (especially echocardiography), limiting its use in emergencies and routine care. These limitations underscore the need for careful patient selection, optimized protocols, and expert interpretation to maximize CMR diagnostic and prognostic value.

## 8. Future Perspectives

Future research in CMR should focus on advanced tissue characterization techniques, including refinements in T1 and T2 mapping and ECV quantification to detect subclinical fibrosis and inflammation [110]. Establishing population-specific reference values that correlate more accurately with clinical outcomes is another important step for their wider clinical implementation. Feature tracking is a developing technique in CMR that uses standard cine sequences to quantitatively analyze myocardial motion and deformation. It measures parameters such as strain, strain rate, torsion, and dyssynchrony to assess cardiac function [111]. DTI, which maps myocardial fiber architecture, holds promise for identifying early microstructural changes linked to arrhythmic risk and HF development [112]. Four-dimensional (4D)-flow CMR can help understand flow behaviour inside the cardiac chambers [112]. Quantitative assessment of the scar and fibrosis burden aims to standardize LGE quantification for better risk stratification and integrate hybrid imaging models, such as combining CMR with positron emission tomography, to enhance predictive accuracy for complex conditions [112]. Advances in low-field magnetic resonance imaging systems can potentially widen access to CMR imaging, especially for obese patients. Machine learning and artificial intelligence are being developed to automate CMR data interpretation, potentially reducing variability and improving efficiency [113]. Artificial intelligence-driven predictive models could personalize risk assessment by incorporating genetics and biomarkers, helping to forecast adverse events and guide clinical decisions. CMR’s potential for screening in high-risk individuals is another research direction, and its effectiveness should be evaluated for early detection of myocardial changes and longitudinal monitoring of selected patients with chronic conditions. Integrating CMR with omics data and other imaging modalities, such as echocardiography, computed tomography, or positron emission tomography, could create comprehensive risk profiles, improving early diagnosis and individualized care. These advancements in CMR may enhance risk stratification, preventive strategies, and development of precision medicine in cardiology.

## 9. Conclusions

Abnormalities in a range of CMR parameters, including LVM, LVEF, LGE, T1 and T2 mapping, and myocardial strain, have been associated with MACE. However, they are not without limitations. The usage of the optimal LVM indexing method is important because it may affect LVH diagnosis, management, and cardiovascular risk assessment. Moreover, it should be taken into consideration that the type and extent of LVH, as well as local functional LV abnormalities, also have prognostic value and may improve assessment of the risk as well as suggest specific etiology. Novel cardiac imaging techniques hold promise in improving the prediction of SCD and the progression of HF and refining strategies for cardiovascular implantable electronic device placement. Future studies are required to standardize novel CMR techniques, define myocardial features that most accurately predict cardiovascular events, and assist in clinical decisions for cardiovascular interventions. Precise data from CMR could be incorporated into clinical decision-making algorithms in different groups of patients and could improve personalized approaches to cardiovascular risk assessment.

## Figures and Tables

**Table 1:** Selected clinical conditions in which left ventricular hypertrophy is valuable in diagnostics and risk stratification.

Clinical Condition	Significance in Diagnostics	Prognostic Implications
Hypertension	Helpful in diagnostics; often correlates with chronically elevated blood pressure	Indicates increased risk for heart failure development; importance of left ventricular geometric pattern
Aortic stenosis	LVH reflects pressure overload	Poor prognosis if untreated severe aortic stenosis; surgery may improve outcomes
Hypertrophic cardiomyopathy	Severe or concentric LVH as well as other CMR features may suggest specific etiologies to be considered; genetic testing may be of value especially in borderline cases; treatment depends on the specific disease	Prognosis varies; risk of sudden cardiac death may depend on severity and extent of LVH
Ischemic heart disease	May develop LVH due to chronic pressure overload, LVH increases myocardial oxygen demand	Associated with higher mortality rates
Athlete’s heart	LVH due to intensive, chronic physical activity	Usually benign; requires differentiation from pathological causes of LVH

Abbreviations: LVH—left ventricular hypertrophy.

**Table 2:** Simplistic comparison of scaling methods used for indexing of left ventricular mass [34,40,41,42].

Compared Feature	Ratiometric Scaling Method	Allometric Scaling Method
Relationship	Linear	Exponential
Scaling equation	LVMi = LVM/body size variable	LVMi = LVM/(body size variable)^scaling exponent^
Examples of used variables and scaling exponents	LVM/BSA LVM/height	LVM/height^1.7^ LVM/height^2.7^ observed/predicted LVM (100 × LVM/[a × height^0.54^ × weight^0.61^] where a = 6.82 (F) or 8.25 (M)
Disadvantages	Scaling a three-dimensional variable (LVM) to a two-dimensional variable (BSA) or one-dimensional variable (height) via simple division is agreeable with the geometric relationship between the two variables.	Complex calculations in everyday clinical practice. Some of them did not fully account for the associations of these measures with body size.
Advantages	Simple calculations in everyday clinical practice.	Demonstrates good correlation between body size and cardiovascular variables that accommodate various relative geometries (eliminates the effects of body size on cardiovascular structure and function).

Abbreviations: BSA—body surface area; F—female; LVM—left ventricular mass; LVMi—left ventricular mass indexing; M—male.

**Table 3:** Selected main studies investigating an indexed left ventricular mass during cardiac magnetic resonance imaging in relatively healthy populations.

Study	Group Characteristics	CMR Methodology	Methods of LVM Indexation	Definition of LVH, Mean Values of LVM, or Percentile Distribution	Studied Relationship Between LVM or LVMi and CVE HR (95% CI)
Luu et al., 2020 [47]	N = 3206 participants free of cardiovascular disease	SSFP sequences	LVM/BSA	Mean ± SD: 61 ± 10 g (M); 48 ± 8 g (F) Normal range: 42–80 g (M); 33–62 g (F) Additionally, they derived LVM reference values indexed to BSA for females and males age 35 to 75 years, stratified by 10-year age categories.	-
Petersen et al., 2017 [38]	N = 800 participants from United Kingdom Biobank (normal healthy Caucasian participants)	SSFP sequences	LVM/BSA	LVH if LVM/BSA > 72 g/m^2^ (M) or >55 g/m^2^ (F)	-
Chuang et al., 2014 [46]	N = 685 participants from Framingham Heart Study (healthy reference group without cardiovascular disease, hypertension, or LV wall motion abnormality)	SSFP sequences	LVM/BSA	P95: 64/44 g/m^2^ (M/F)	-
			LVM/height	P95: 74/49 g/m (M/F)	
			LVM/height^2.7^	P95: 29/22 g/m^2.7^ (M/F)	
Jain et al., 2011 [42]	N= 4965 participants from MESA study (multiethnic cohort free of clinically recognized CVD at enrollment)	GRE sequences	Observed/predicted LVM	-	CHD diagnosis: 1.1 (1.0–1.3) ‡ Stroke: 1.3 (1.1–1.7) ‡ and 1.3 (1.1–1.7) § HF diagnosis: 1.8 (1.6–2.1) ‡ and 1.8 (1.6–2.1) § All CVD events: 1.4 (1.2–1.5) ‡ and 1.3 (1.2–1.5) §
Chirinos et al., 2010 [34]	N = 523 participants from MESA study (multiethnic cohort free of clinically recognized CVD at enrollment)	GRE sequences	LVM/height^1.7^ LVM/height^2.7^ LVM/BSA	Normalized LVM percentile values in Asklepios and MESA reference samples	Cox models assessing the performance of LVM indexed for height^1.7^, height^2.7^, and BSA predicting CVE (defined as myocardial infarction, resuscitated cardiac arrest, coronary heart disease death, stroke, or stroke death, angina, other atherosclerotic death, or other cardiovascular disease death) and all-cause-death in MESA reference sample
Brumbarck et al., 2010 [41]	N = 822 participants from MESA study (multiethnic cohort free of clinically recognized CVD at enrollment)	GRE sequences	ppLVmassH	1.33 (1.29, 1.37)	CVE (combined nonfatal and fatal coronary heart disease and stroke): 2.1 (1.4–3.1) †
			ppLVmassHW	1.31 (1.28, 1.36)	CVE (combined nonfatal and fatal coronary heart disease and stroke): 2.4 (1.5–4.0) †
			LVM/BSA	106.2 (103.8, 110.9) g/m^2^ (M) and 84.6 (82.2, 88.3) g/m^2^ (F)	CVE (combined nonfatal and fatal coronary heart disease and stroke): 2.2 (1.4–3.4) †
			LVM/height^2^	65.7 (62.3, 68.0) g/m^2^ (M) and 53.0 (51.1, 55.3) g/m^2^ (F)	CVE (combined nonfatal and fatal coronary heart disease and stroke): 2.0 (1.4–3.0) †
			LVM/height^2.7^	45.1 (42.7, 47.0) g/m^2.7^ (M) and 38.0 (36.5, 39.5) g/m^2.7^ (F)	CVE (combined nonfatal and fatal coronary heart disease and stroke): 2.1 (1.4–3.2) †
Bluemke et al., 2008 [16]	N = 4968 participants from MESA study (multiethnic cohort free of clinically recognized CVD at enrollment)	GRE sequences	LVM/LV volume in quartiles (4th quartile)	-	CHD diagnosis: 5.3 (2.9–10.0) and 2.3 (1.2–4.4) * Stroke: 23.0 (3.1–170.5) and 11.1 (1.4–84.8) *
			Observed/predicted LVM (≥95th percentile)	-	HF diagnosis: 13.0 (6.1–27.7) and 8.6 (3.7–19.9) *
Natori et al., 2006 [48]	N = 800 participants from MESA study (multiethnic cohort free of clinically recognized CVD at enrollment)	GRE sequences	LVM/BSA	85.1 ± 15.2 g/m^2^ (M) and 66.9 ± 10.9 g/m^2^ (F);	-
			LVM/BMI	6.32 ± 1.21 g/kg/m^2^ (M) and 4.39 ± 0.82 g/kg/m^2^ (F);	
			LVM/height	0.94 ± 0.19 g/cm (M) and 0.71 ± 0.14 g/cm (F);	
			LVM/weight	2.11 ± 0.38 g/kg (M) and 1.72 ± 0.30 g/kg (F).	

Abbreviations: CI—confidence interval; CMR—cardiac magnetic resonance; CVD—cardiovascular disease; CVE—cardiovascular event; GRE—gradient echo; HF—heart failure; MESA—the Multi-Ethnic Study of Atherosclerosis; P95—indicates the upper 95th percentile limit; ppLVmassH—percent-predicted LV mass based on height and sex; ppLVmassHW—percent-predicted LV mass based on height, weight, and sex; SSFP—steady-state free precession. For other abbreviations, see Table 2. * Adjusted for the following risk factors: age, gender, race, cigarette smoking, total cholesterol, HDL cholesterol, use of lipid lowering medication, systolic blood pressure, diastolic blood pressure, use of anti-hypertensive drugs and diabetes; † Adjusted for age and gender; ‡ Adjusted for traditional risk factors (age, sex, ethnicity, BMI, systolic blood pressure, total and HDL cholesterol, diabetes, cigarette smoking, hypertension, and lipid medication); § Adjusted for traditional risk factors as well as imaging-derived measures (CAC, IMT, LVM, and LVM/volume) in the same model.

**Table 4:** Selected studies describing associations between different cardiac magnetic resonance parameters and cardiovascular events.

Study	Characteristics of Patients	Study Design	Evaluated Parameters	Assessed Cardiovascular Events	Associations of Evaluated Parameters with Cardiovascular Events
Weng et al. (2016) [67]	N = 2993 with HCM	Meta-analysis	Any presence and extent of LGE	SCD, all-cause mortality, cardiovascular mortality, HF death	Any presence of LGE: SCD (OR: 3.41; 95% CI: 1.97–5.94; *p* < 0.001) All-cause mortality (OR: 1.80; 95% CI: 1.21 to 2.69; *p* = 0.004) Cardiovascular mortality (OR: 2.93; 95% CI: 1.53 to 5.61; *p* = 0.001), HF death (OR: 2.21; 95% CI: 0.84 to 5.80; *p* = 0.107). Extent of LGE: SCD (HR: 1.56/10%; 95% CI: 1.33–1.82; *p* < 0.0001) HF death (HR: 1.61/10% LGE; 95% CI: 1.21–2.13; *p* = 0.001) All-cause mortality (HR: 1.29/10% LGE; 95% CI: 1.09–1.51; *p* = 0.002) Cardiovascular mortality (HR: 1.57/10% LGE; 95% CI: 1.30–1.89; *p* < 0.001).
Briasoulis et al. (2015) [62]	N = 3067 with HCM	Meta-analysis	Any presence of LGE	SCD	SCD (OR: 2.52; 95% CI: 1.44–4.4; *p* = 0.001)
Green et al. (2012) [61]	N = 1063 with HCM	Meta-analysis	Any presence of LGE	Cardiac death, HF death, all-cause mortality	Cardiac death (pooled OR: 2.92; 95% CI: 1.01–8.42; *p* = 0.047), HF death (pooled OR: 5.68; 95% CI: 1.04–31.07; *p* = 0.045), All-cause mortality (pooled OR: 4.46; 95% CI: 1.53–13.01; *p* = 0.006)
Mordi et al. (2015) [88]	N = 539 consecutive patients referred for clinically indicated CMR who underwent a CMR protocol that included cine imaging, tagging, and LGE	Prospective cohort	LVEF, presence of LGE (5SD), and reduced GCS	Prevalence of MACE (a composite of all-cause mortality, HF-related hospitalization, and aborted SCD)	LVEF (%): (HR: 0.96; 95% CI: 0.94–0.99; *p* = 0.005), Presence of LGE (5SD): (HR: 2.07; 95% CI: 1.03–4.14; *p* = 0.04), Reduced GCS (%): (HR: 1.11; 95% CI: 1.02–1.21; *p* = 0.04).
Pezel et al., 2021 [89]	N = 31,762 consecutive patients referred for stress CMR	Prospective cohort	Inducible ischemia and any presence of LGE	Mortality	Inducible ischemia: (HR, 1.61; 99.5% CI: 1.41–1.84; *p* < 001), Any presence of LGE: (HR: 1.62; 99.5% CI: 1.41–1.86; *p* < 0.001).
Banypersad et al., 2015 [76]	N = 100 consecutive patients with systemic amyloid light-chain amyloidosis	Retrospective cohort	T1 values > 1044 ms ECV values > 45%	Mortality	ECV > 45% (HR: 3.84; 95% CI: 1.53–9.61; *p* = 0.004) Pre-contrast T1 of >1044 ms (HR: 5.39; 95% CI: 1.24–23.4; *p* = 0.02)
Vita et al., 2019 [77]	N = 240 patients with nonischemic dilated cardiomyopathy	Retrospective cohort	LVEF, any LGE presence, mean ECV	Death from any cause or HF decompensation requiring hospitalization.	LVEF (%) (HR: 0.94; 95% CI: 0.92–0.97; *p* < 0.001) Any LGE presence (HR: 2.26; 95% CI: 1.16–4.40; *p* = 0.016), Mean ECV (%) (HR: 1.11; 95% CI: 1.07–1.15; *p* < 0.0001).
Mileva et al., 2023 [84]	N = 770 patients with suspected MINOCA	Meta-analysis	CMR diagnosis	MACE (cardiovascular death, nonfatal MI, and HF hospitalization) occurrence	CMR diagnosis of MINOCA (pooled OR: 2.40; 95% CI: 1.60–3.59). Diagnoses of myocarditis and takotsubo syndrome were not significantly associated with increased risk of combined clinical outcomes).
Schumm et al., 2014 [82]	N = 405 patients with suspected myocarditis	Prospective cohort	The presence of LGE, LVEF and LVEDV	Primary endpoint (cardiac mortality and MACE, including cardiac death, SCD, aborted SCD, and appropriate ICD discharge) Secondary endpoint (composite of primary endpoint and hospitalization for HF).	Primary endpoint LVEF (per % increase): HR: 0.939; *p* = 0.01. LVEDV (per mL increase): HR: 0.999; *p* = 0.87. LGE: HR: 3.98; *p* = 0.11. Endpoint 2 LVEF (per % increase): HR: 0.965; *p* = 0.03. LGE: HR: 2.919; *p* = 0.02. LVEDV (per mL increase): HR: 1.002; *p* = 0.33.
Bergamaschi et al., 2024 [85]	N = 171 consecutive patients with STEMI	Prospective cohort	T2 values	Primary endpoint (MACE, defined as cardiovascular death, MI, unplanned coronary revascularization or rehospitalization for HF).	Higher non-infarcted myocardium T2 values independently predicted MACE (HR: 2.824; 95% CI: 1.254–6.361; *p* = 0.012).
Carrick et al., 2016 [86]	N = 300 reperfused STEMI patients (n = 160 patients with a native T1 infarct core)	Prospective cohort	Native T1 values within the hypo-intense infarct core	All-cause death or first hospitalization for HF post-discharge.	For a 10 ms increase in native T1: HR: 0.730; 95% CI: 0.617–0.863; *p* < 0.001) including after adjustment for LVEF, infarct core T2, and myocardial haemorrhage.

Abbreviations: CI—confidence interval; CMR—cardiac magnetic resonance; GCS—global circumferential strain; HCM—hypertrophic cardiomyopathy; HF—heart failure; HR—hazard ratio; LGE—late gadolinium enhancement; LVEF—left ventricular ejection fraction; MACE—major adverse cardiovascular events; MI—myocardial infarction; MINOCA—myocardial infarction with non-obstructive coronary arteries; OR—odds ratio; SCD—sudden cardiac death; STEMI—ST-segment elevation myocardial infarction; VA—ventricular arrhythmias.

## Data Availability

Not applicable.
